# Quantifying traffic noise pollution levels: a cross-sectional survey in South Africa

**DOI:** 10.1038/s41598-022-07145-z

**Published:** 2022-03-02

**Authors:** Nomfundo Moroe, Paballo Mabaso

**Affiliations:** Department of Speech Pathology and Audiology, University of the Witwatersrand, Johannesburg, South Africa

**Keywords:** Environmental sciences, Environmental social sciences

## Abstract

Despite the alarming increase in environmental noise pollution, particularly road traffic noise, in developing countries, there seems to be no awareness regarding the long-term impacts of noise, specifically traffic noise, on the health outcomes of individuals exposed to excessive noise. Additionally, there is a dearth of studies on noise and its effects utilising the pollution modelling technique known as Pollution Standard Index (PSI) to analyse the impact of noise pollution on exposed individuals. The aim of this study was to investigate the noise levels commuters are exposed to and to apply PSI to determine the level of exposure. We conducted a cross-sectional study at two taxi ranks, over 28 days. Eighty-four noise measurements were collected using a sound level meter and a dosimeter at different times of the day and month, peak vs off-peak hours and busy days vs quiet days. Data were collected between April and July 2019. We used the Pollution Standard Index to analyse the data. Noise levels were above the permissible commercial noise levels as they fell within the extremely dangerous noise sensitivity zone as determined by the PSI. Furthermore, the noise levels fell below the WHO maximum permissible level of 90 dB. There was no statistical difference between the means of the open and closed ranks. Dosimeter noise level recordings fell within the satisfactory zone as measurements were below 300 PSI, which is considered unhealthy. There is a need to raise awareness on the dangers and effects of noise pollution in developing countries, as their populations are exposed to road traffic noise.

## Introduction

Noise is an ubiquitous environmental hazard of the modern world, emanating from a wide variety of sources, which contribute immensely to environmental noise pollution^1^. The World Health Organization (WHO) recognises noise pollution—defined as unfavorable noise caused by human activity^2^—as a serious public health issue^3^. Noise pollution is considered the third most harmful factor in large cities^4^. As such, the Occupational Safety and Health Association (OSHA) has set 90 dBA as the time-weighted average (TWA) for an 8-hour workday exposure to noise, while the National Institute of Occupational Safety and Health (NIOSH) sets the limit at 85 dBA^5^.

Noise pollution in urban areas is caused by a variety of sources such as road traffic, construction, businesses, airports, and industrial and residential areas; vehicle traffic contributes the most to the production of urban noise^4^ and results in physiological effects that subsequently contribute to a large burden of disease^6^. Globally, traffic noise is a major source of environmental pollution^7^, and conservatively, it is estimated that one million healthy life years are lost every year to traffic related noise in the western part of Europe^6^. In Sweden, the number of DALYs (disability adjusted life years) attributed to traffic noise is estimated to be 41,033 years^8^, while in Germany, a total of 26,501 DALYs is attributed to road traffic noise^9^. In developing countries, such data is not readily available. This may be due to the lack of quantified noise levels in these places.

Transportation is an essential part of modern society; however, its benefits may be overshadowed by its negative consequences ^10^. While Kreis^6^ argues that noise pollution is comparable to air pollution, Banerjee^10^ contends that road traffic noise is unique in comparison to other pollutants, such as air or water, as noise has no immediate residual evidence to serve as a reminder of its negative consequences, except for an occasional ringing in the ears, known as tinnitus, headaches, and stress. Furthermore, traffic noise has been associated with a range of auditory and non-auditory outcomes including all-cause premature death, cardiovascular death and morbidity, annoyance and sleep disturbances, adverse productive outcomes and increased levels of stress and aggression^6^. According to Khreis^6^, low-income individuals and visible minorities are generally located in areas with high levels of road traffic noise.

Various methods or techniques, such as noise impact index, traffic noise index and noise pollution levels, have been used to quantify noise^11^. For the current study, we used the pollution standard index (PSI) which is a pollution modelling technique based on the daily ambient concentration of pollutants and is categorised into six levels: good (0–50), moderate (51–100), unhealthy for sensitive groups (101–199), unhealthful (200–299), very unhealthful (300–400) and hazardous (over 401)^12^. Few studies have employed this technique. Specific to noise pollution, this technique has been used in Nigeria^12^. The PSI is important because it reports the actual concentrations of each pollutant, their degree of pollution and effects^13^.

There is a dearth of research on noise pollution levels in sub-Saharan Africa, save for a few studies conducted in Nigeria^14–17^. In South Africa, there is a plethora of studies on occupational noise and policies on environmental noise, but there is a dearth of studies on environmental noise pollution. This is a concern considering that South Africa is one of the most urbanised countries in sub-Saharan Africa. Furthermore, the majority of the working population in South Africa relies on public transport to commute between work and home on a daily basis. Potentially, these people are exposed to environmental noise levels above the permissible legislation of 85 dB(A) as recommended by NIOSH^5^. South Africa is experiencing a rapid population growth with an increasing demand for travel. As such, there is a heavy reliance on public transport to gain access to economic, social, educational, medical, recreational and cultural activities^18^. Reportedly, in 2013, 91.4% of people in the lowest income group in South Africa relied on public transport, with 5.7% using trains, 23.6% buses and 62.1% minibus taxis^18^. Based on the figures above, it is evident that road traffic noise is prevalent in the lowest income group. To put this into perspective, in 2014, a survey to assess the use of public transport in South Africa revealed that only 30% of households own a car with 70% relying on taxis, buses, trains and other non-motorised transport modes^19^.

In view of the alarming increase in environmental noise pollution, most countries such as Australia, India, Japan, and the United States have permissible noise levels^10^. For instance, the United States Federal Highways Administration and the World Health Organization have noise standards for various land uses including residential, commercial, industrial, etc.^20^ Additionally, the United State Environmental Protection Agency also has noise sensitivity zones as depicted in Table 1.

**Table 1:** Noise sensitivity index (Source^10^).

dB (A)	Sensitivity
55–< 60	Risky
60–< 65	Moderately risk
65–< 70	Highly risk
70–< 75	Dangerous
75–< 80	Highly dangerous
> 80	Extremely dangerous

Similarly, in South Africa, there are regulations pertaining to ambient noise levels as espoused in the City of Tshwane Noise Management Policy^21^. According to this policy, in urban districts—specifically some workshops, business premises as well as main roads—noise levels should not exceed 60 dB(A) in an outdoor setting and 50 dB(A) in an indoor (with windows) setting. These values are for the day-night period. While these regulations are available, there is no evidence of their implementation or enforcement. In the absence of enforcement of these regulations, there is a need to highlight the immediate and latent effects of environmental noise on the general public. Already, South Africa, as a developing country, is faced with a quadruple burden of disease, low levels of education and employment^22–24^. As such, this country cannot afford to add to these challenges. In fact, in line with the World Health Organization, there is a need to target primary prevention, by raising awareness of the effects and the dangers associated with traffic noise to the unsuspecting population. Such efforts will benefit from evidence-based recommendations, such as noise level measurements, in places like taxi ranks, where most traffic noise is potentially produced. Therefore, the aim of this study was to measure environmental noise levels at two taxi ranks in South Africa. This was achieved by measuring environmental noise using a sound level meter as well as a dosimeter to determine personal exposure.

## Subjects and methods

### Sample

A cross-sectional study was carried out at two taxi ranks in Johannesburg. These two ranks were chosen because they are the busiest and biggest taxi ranks in Johannesburg. Johannesburg is in Gauteng Province, and it is the smallest, yet  most overpopulated province in South Africa. It is the country’s economic hub. Taxi Rank A is an open one-story building with openings on all sides of the building, supported by approximately 40 pillars. It is situated in the heart of Soweto—one of the biggest townships in South Africa. It is separated by a fence from the continent’s largest and the third-largest hospital in the world^25^. This rank stretches over 1.3 kms, with a width of 50 m^26^. It accommodates at least 500 taxis in holding bays, with 160 taxi loading bays, 35 long-distance taxi loading bays and 20 bus bays with approximately 500 traders and 550,000 commuters^25^.

Taxi Rank B is a closed rank, meaning taxis are housed in an enclosed three-story building with four sides closed and four garage door openings, two on either side of the building. These garage door openings serve as entrances and exits for taxis and commuters. It is designed to accommodate at least 25 buses serving 35 different routes, 800 traders, 500,000 commuters daily and at least 3,000 to 4,000 taxis^26^.

### Ethics approval

Ethics clearance was obtained from the University’s Human Research Ethics Committee (HREC) (non-medical) of the University of the Witwatersrand, Johannesburg, South Africa, Protocol Number: STA_2019-04. Additionally, methods followed in this study were performed in accordance with the relevant guidelines and regulations as stipulated by the University of the Witwatersrand Ethics Committee. Lastly, this study did not include any human participants.

### Data collection tools

This study was conducted over 28 days from 27 April to 10 May 2019 at Rank A and 11 May to 09 July 2019, with a break between 13 May and 21 June 2019 at Rank B. Data were collected in three intervals: 6:00–8:30; 11:00–13:30 and 16:30–18:30. These times were targeted to include both peak (morning and afternoon) and off-peak (mid-morning) traffic. All noise measurements were conducted by the same person using a sound level meter and a dosimeter.

### Sound level meter

Noise measurements were conducted using the Quest technologies’ 210 sound level meter (SLM). At each rank, and with each recording, the SLM was mounted on a tripod at a height of a metre from the ground. Noise levels were conducted at 30-min intervals at the middle or centre of the rank, at the back of the rank as well as the back of the ground floor. In Rank B, the ground floor was chosen because most of the activities take place on this floor. The recordings were conducted over 28 days, 14 in each taxi rank. The recordings were done for daytime only (from 6:00 to 18:30) for safety purposes. In total, 84 readings were taken from both ranks.

### Dosimeter

A dBadge2 Casella dosimeter was used to collect and measure personal exposure noise levels. A dosimeter was clipped on the lapel of the researcher’s clothing. The dosimeter was set to the A-weighting network which is the most common frequency weighting for environmental and industrial studies. The dosimeter was then set on automatic mode to run continuously for 30 min at every instance. After each interval, the dosimeter calculated the average noise the researcher was exposed to and recorded it as an average noise level or equivalent noise level. Furthermore, the researcher collected traffic noise by commuting on short journeys (routes) to sample traffic noise. In total, 28 recordings were taken over 28 days.

### Data analysis

Sound level meter data were analysed using descriptive statistical analyses, which included simple line graphs to summarise the data, while inferential statistics were analysed in the form of a one-way analysis of variance (ANOVA) and a two-sample t-test. Dosimeter measurements were analysed using descriptive statistics, power analysis for correlation as well as the pollution modelling technique known as Pollution Standard Index (PSI). The use of the Pollution Standard Index (Equation 1) was applied to better appreciate the environmental noise position in the given area. This modelling is premised on a function $\alpha$, where $\alpha$ is ascribed a number indicating the quality standard. This modelling approach considers the weighted values of individual pollutant parameters measured at spatial points, which is then compared to the single number of the quality standard as presented in Table 2.

**Table 2:** Standard Index for noise measurements incorporating PSI values as well as equivalent noise levels.

PSI values	Index category	Equivalent noise levels (dBA)	Noise description
< 100	Very good quality	20	Very quiet
100	Good quality	50	Quiet
200	Satisfactory	70	Noisy
300	Unhealthy	90	Very noisy
400	Hazardous	100	Unbearable
500	Seriously hazardous	130	Seriously hazardous

The attraction of the model is that the number $\alpha$ is a non-dimensional number^27^. According to Kiely^28^, the non-dimensional equation can be obtained as:

1 $$\alpha = \alpha_{{\text{i}}} + \frac{{\alpha_{{{\text{i}} + 1}} - \alpha_{1} }}{{{\text{C}}_{{{\text{i}} + 1}} - {\text{C}}_{{\text{i}}} }}\left( {{\text{C}} - {\text{C}}_{{\text{I}}} } \right)$$

where $\alpha$ = pollutant standard index, C = corresponding pollutant concentration, α_1 =_ breakpoint PSI from one quality to another. (Equation 1)

## Results

### Sound level meter measurements

#### Rank A (Open)

**Figure 1:**
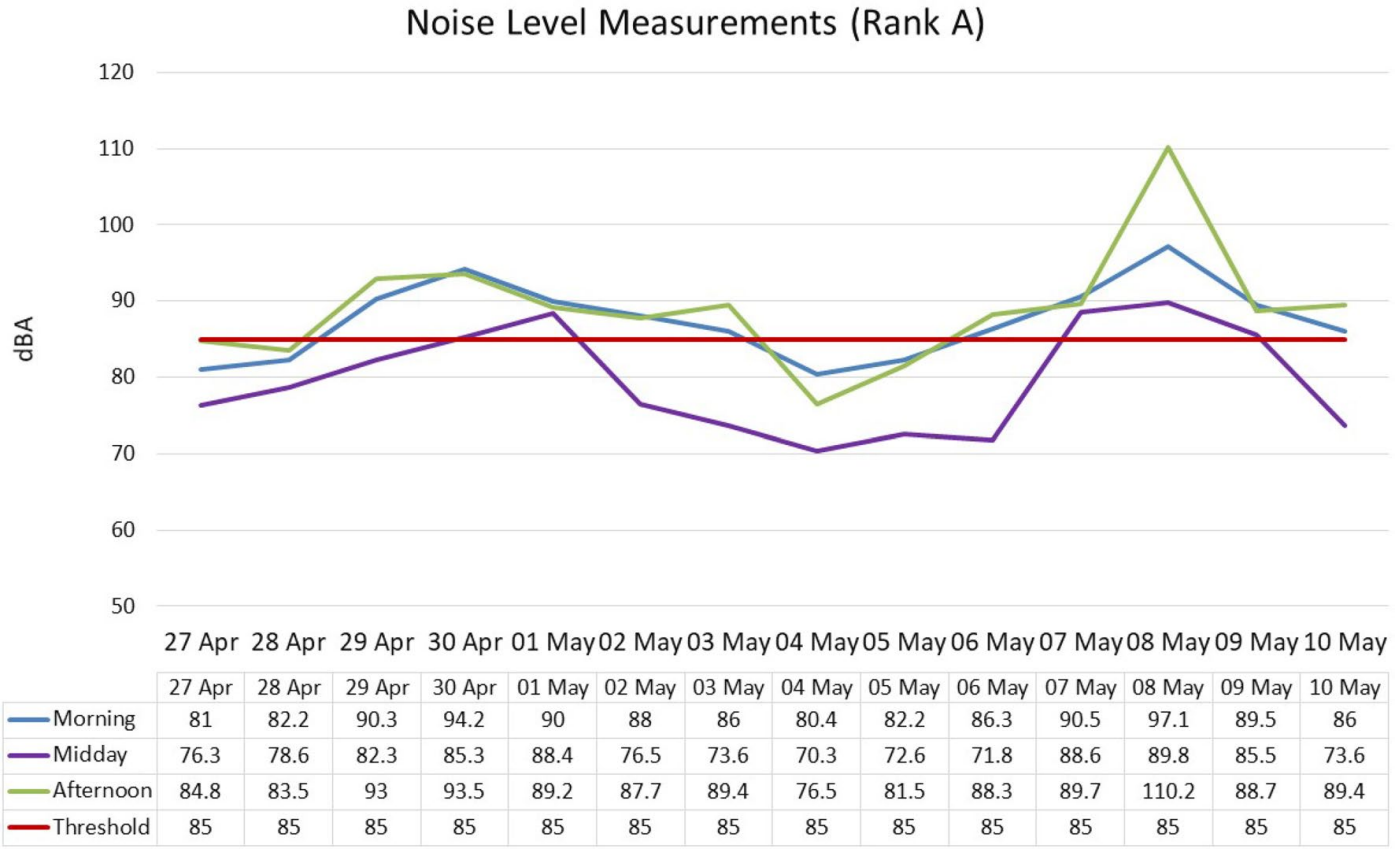
Noise level measurement: Rank A (Open).

The mean value levels in Rank A, regardless of the period of the day was 85.3 dB(A), ranging from 70.3 to 110.2 dB(A), (**± **SD 7.7). When analysed according to period of the day, morning—87.40 dB(A), (**± **SD 4.95); mid-morning—79.51 dB(A) (**± **6.9 SD); and afternoon—88.9 dB(A) (**± **SD 7.6) (Fig. 1). Respectively, the noise levels ranged from 80.4 dB(A) to 97.1 (morning); 70.3 dB(A) to 88.6 dB(A) (mid-morning); and 76.5 dB(A) to 110.2 dB(A) (afternoon). The results of the ANOVA (Table 3) revealed a statistically significant difference between the periods of the day (independent variables) and noise levels (dependent variable), *p* = 0.0010.

**Table 3:** Analysis of variance of noise levels for morning, midday and afternoon.

ANOVA					
Source	Sum of squares	df	Mean square	F	Sig.
Between groups	718.047143	2	359.023571	8.26	0.0010
Within groups	1695.02071	39	43.4620696		
Total	2413.06786	41	58.8553136		

*Df* degree of freedom, *Sig.* level of significance.

#### Rank B (Closed)

**Figure 2:**
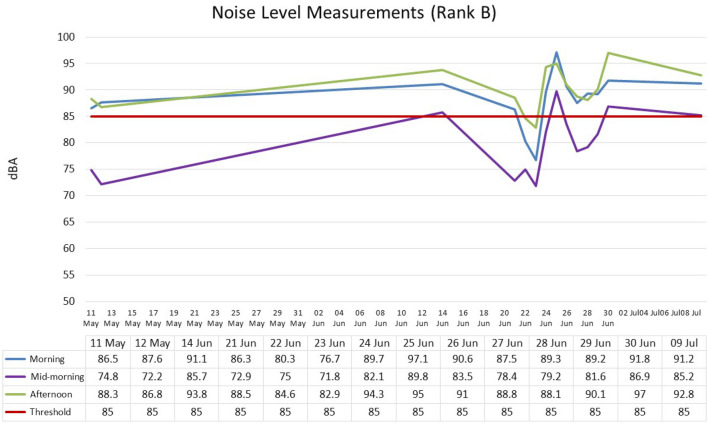
Noise level measurements: Rank B (Closed).

Regardless of the time of day, the mean value level at Rank B was 86.1 dB(A) and ranged from 71. dB(A) to 97 dB(A) (± SD 6.7). Figure 2 shows mean value levels according to different periods of the day: morning—88.21 dB(A) (± SD4.97); mid-morning—79.93 dB(A) (± SD 5.9); and afternoon—90.14 dB(A) (± SD 4.07). The minimum and maximum noise levels were recorded at 71 dB(A) and 97 dB(A) respectively. These noise levels ranged from 76.7 to 97.1 dB(A) (morning); 71.8 dB(A) to 98.8 dB(A) (midday);  and 82.9 dB(A) to 97 dB(A) (afternoon), respectively. Similarly, for Rank B, the ANOVA test (Table 4) revealed a statistically significant difference between variables *p* = 0.0000.

**Table 4:** Analysis of variance of noise levels for morning, midday and afternoon.

ANOVA					
Source	Sum of square	df	Mean square	F	Sig.
Between groups	822.963333	2	411.481667	16.15	0.0000
Within groups	993.375714	39	25.4711722		
Total	1816.33905	41	44.3009524		

*Df* degree of freedom, *Sig.* level of Significance.

### Comparison of the average noise levels in Rank A and Rank B according to time of day

**Figure 3:**
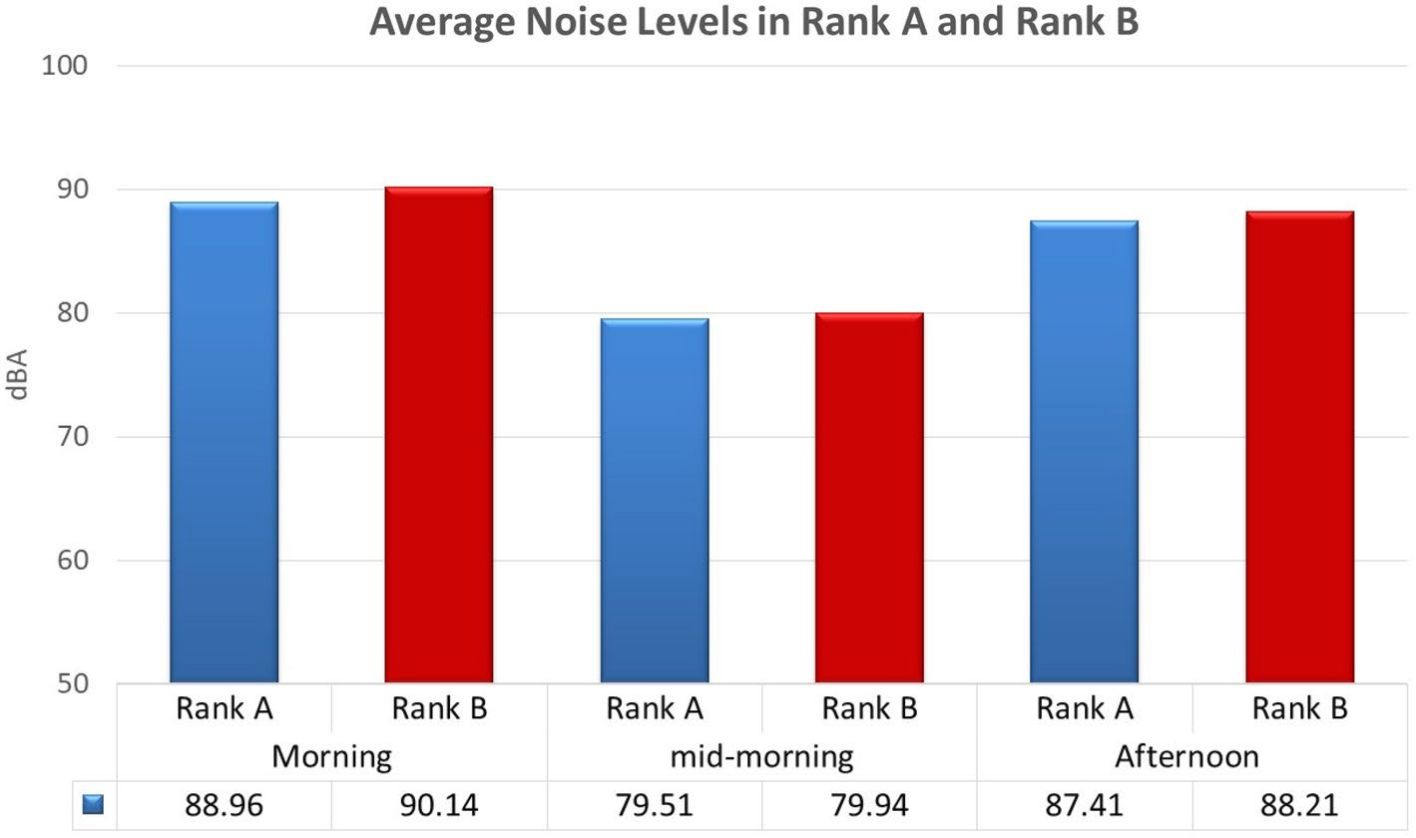
Average noise levels in Rank A and Rank B.

Figure 3 shows that the mean value level at Rank A was 88.96 dB(A) and 90.14 dB(A) for Rank B in the morning; 79.51 dB(A) for Rank A and 79.94 dB(A) for Rank B mid-morning; and 87.41 dB(A) for Rank A and 88.21 dB(A) in the afternoon. Similarly, a two-sample t-test with equal variance was conducted to compare the mean of both ranks, regardless of the time of day. The overall mean noise level recorded in Rank A was 85.3 (± SD 7.7) compared to 86 dB(A) (± SD 6.6). Statistically, the two-sample t-test revealed no statistically significant differences between the means of the two ranks across the day *p* = 0.6100 (Table 5*).* Similarly, the analysis of the means, taking into account the different times of day, revealed no statistical difference according to the period of the day as the values were *p* = 0.6100 (morning); *p* = 0.8642 (mid-morning) and *p* = 0.6734 (afternoon). Therefore, it can be concluded that there is no statistical difference in the noise levels from both ranks.

**Table 5:** Two-sample t-test with equal variances ƒ.

Two-sample t-test with equal variances						
Groups	Observations	Mean	Std err	Std dev	95% Conf interval	
Rank A	42	85.29286	1.183772	7.671722	82.90218	87.68353
Rank B	42	86.09524	1.027027	6.655896	84.02111	88.16936
Combined	84	85.69405	.7801069	7.149797	84.14245	87.24565

The noise level readings for both ranks were further analysed according to the noise sensitivity zones (Table 6). The summary of the measurements revealed that 83.3% of the total noise levels fell within the extremely dangerous zone (> 80), accounting for 70 of the 84 average noise levels recorded. A total of 6% of the average noise levels fell within the highly dangerous zone of 75–79 dB(A) and 10.7% fell within the dangerous zone of 70–74 dB(A). Based on this analysis, it is evident that the noise levels at these two ranks are above the recommended noise levels limit.

**Table 6:** Percentile distribution of the recorded average noise levels.

Noise level dB (A)	Total	Percentage	Sensitivity
55–< 59	0	0	Risky
60–< 64	0	0	Moderately risky
65–< 69	0	0	Highly risky
70–< 74	9	10.7	Dangerous
75–< 79	5	6	Highly dangerous
> 80	70	83.3	Extremely dangerous

### Dosimeter noise measurements

#### Rank A

**Figure 4:**
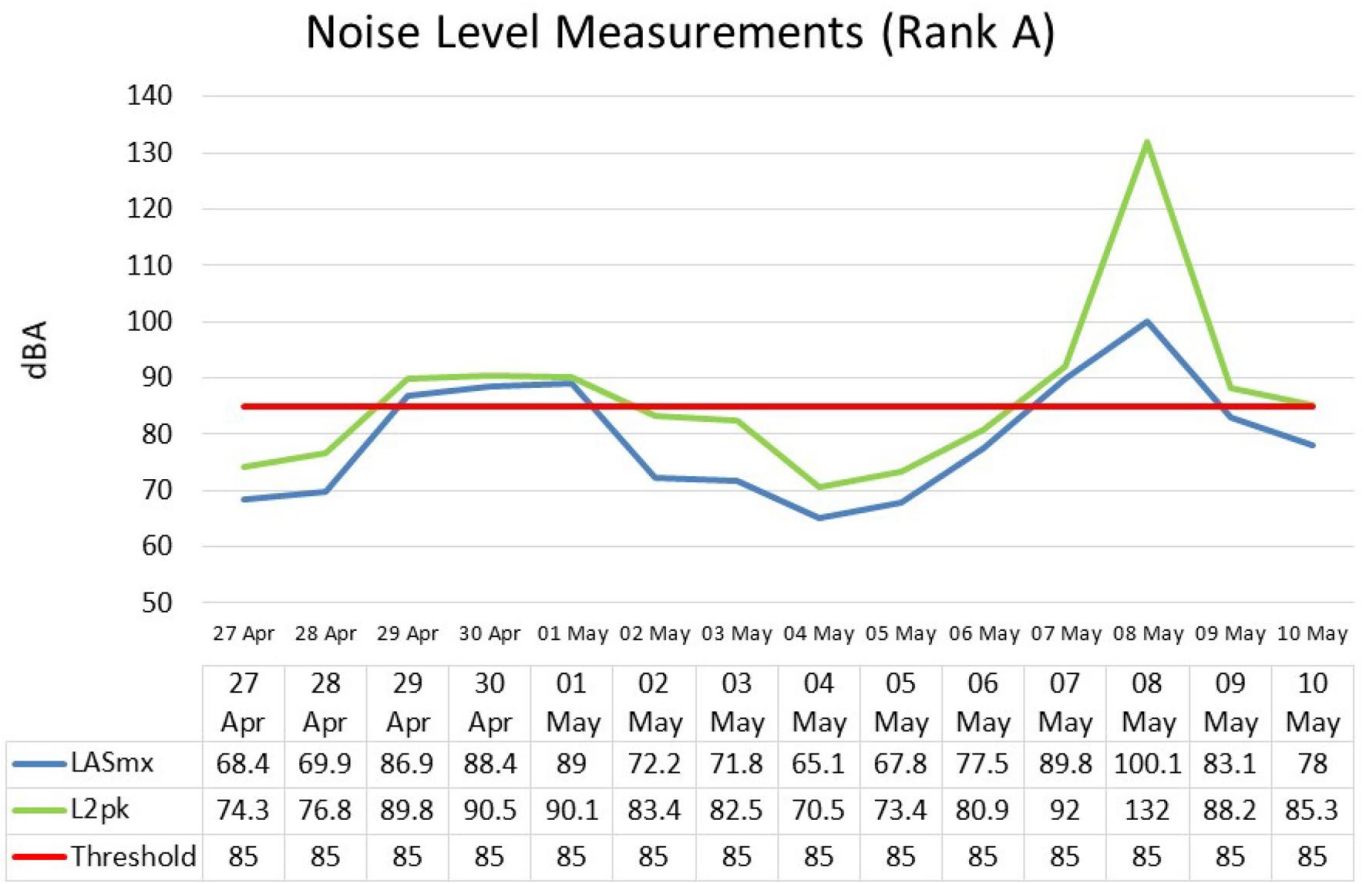
Noise measurements for Rank.

The mean LASmx is 79.1 dB(A), and the noise levels range from 65.1 to 100 dB(A). The mean for L2pk is 86.4 dB(A) and the noise levels range from 70.5 to 132 dB(A) (Fig. 4). The highest noise level was on 8 May with the least exposure on 04 May. In comparing the LASmx and L2pk, there was a positive correlation between the two variables, *r* = 0.87,* p* = 1.0000.

### Rank B

**Figure 5:**
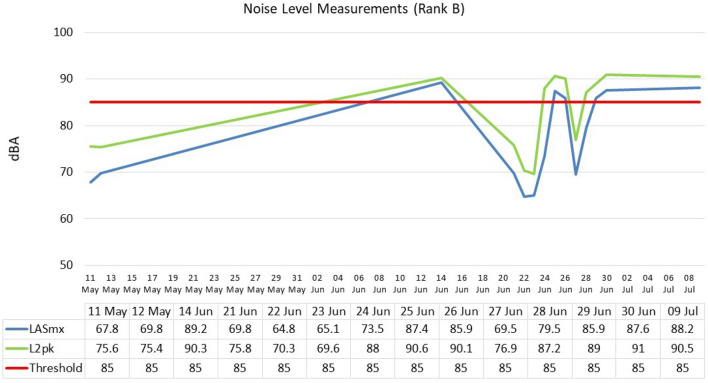
Noise measurements in Rank B.

The mean LASmx is 77.4, and the noise levels range from 64.8 to 89.2 dB(A). The mean for L2pk (peak) is 82.9 dB(A), with the noise levels ranging from 69.6 to 91 dB(A) (Fig. 5). The maximum exposure was collected on 25 June with the least exposure on 04 June. In comparing the LASmx and L2pk, there was a positive correlation between the two variables*, r* = 0.94,* p* = 1.0000.

### The analysis of both ranks

In comparing the LASmx and L2pk for both ranks, there was a positive correlation between the variables, *r* = 0.87,* p* = 1.0000. The noise measurements for LASmx and L2pk (Table 7) were further compared with the NIOSH standard of permissible noise levels (85 dBA)(5). From these results, the equivalent noise level as well as the peak measurements  fell below the NIOSH maximum standard of 85 dB for most days, except for 29 and 30 April and 01 May; 07 and 08 May which were above the recommended standard. Similarly, the majority of the L2pk (Table 8) noise levels fell within the permissible levels except for 14, 25, 26, 29, 30 June, and 09 July.

**Table 7:** Noise measurements recorded at Rank A.

Day	Equivalent noise level (Leq) (dB)	Leq against NIOSH standard (%)	Peak noise level (dB)	Peak against NIOSH standard (%)
27 Apr	68.4	80.5	74.3	87.4
28 Apr	69.9	82.2	76.8	90.4
29 Apr	86.9	102.2	89.8	105.6
30 Apr	88.4	104.0	90.5	106.5
01 May	89	104.7	90.1	106.0
02 May	72.2	84.9	83.4	98.1
03 May	71.8	84.5	82.5	97.1
04 May	65.1	76.6	70.5	82.9
05 May	67.8	79.8	73.4	86.4
06 May	77.5	91.2	80.9	95.2
07 May	89.8	105.6	92	108.2
08 May	100.1	117.8	132	155.3
09 May	83.1	97.8	88.2	103.8
10 May	78	91.8	85.3	100.4

**Table 8:** Noise measurements recorded at Rank B.

Day	Equivalent noise level (Leq) (dB)	Leq against NIOSH standard (%)	Peak noise level (dB)	Peak against NIOSH standard (%)
11 May	67.8	79.8	75.6	88.9
12 May	69.8	82.1	75.4	88.7
14 Jun	89.2	104.9	90.3	106.2
21 Jun	69.8	82.1	75.8	89.2
22 Jun	64.8	76.2	70.3	82.7
23 Jun	65.1	76.6	69.6	81.9
24 Jun	73.5	86.5	88	103.5
25 Jun	87.4	102.8	90.6	106.6
26 Jun	85.9	101.1	90.1	106.0
27 Jun	69.5	81.8	76.9	90.5
28 Jun	79.5	93.5	87.2	102.6
29 Jun	85.9	101.1	89	104.7
30 Jun	87.6	103.1	91	107.1
09 Jul	88.2	103.8	90.5	106.5

In applying the PSI analysis, the non-dimensional equation (Equation 1), as discussed by Kiely^28^, was utilised. The results obtained are presented in Table 9, while Table 10 indicates the PSI values. According to the results, with the exception of 8 May, the noise levels at both ranks fell within the satisfactory zone as all the values fell below 300 PSI, although some were slightly above 300. Values above 300 are considered unhealthy. The healthy or safe mean for LASmx was 249.27 with L2pk of 282.73 for Rank A. Similarly, Rank B’s mean for LASmx was 237.14 and 265.29 L2pk. There was no statistical difference between the mean for the LASmx and L2pk for both ranks, p = 0.566 (MASmx) and p = 0.453 (L2pk).

**Table 9:** Standard index measurement^10^.

Rank A (open)			Rank B (closed)		
PSI values			PSI values		
Date	LASmx (dB)	L2pk (dB)	Date	LASmx (dB)	L2pk (dB)
27 Apr	192.00	221.50	11 May	189.00	228.00
28 Apr	199.50	234.00	12 May	199.00	227.00
29 Apr	284.50	299.00	14 Jun	296.00	303.00
30 Apr	292.00	305.00	21 Jun	199.00	229.00
01 May	295.00	301.00	22 Jun	174.00	201.50
02 May	211.00	267.00	23 Jun	175.50	198.00
03 May	209.00	262.50	24 Jun	217.50	290.00
04 May	175.50	202.50	25 Jun	287.00	306.00
05 May	189.00	217.00	26 Jun	279.50	301.00
06 May	237.50	254.50	27 Jun	197.50	234.50
07 May	299.00	320.00	28 Jun	247.50	286.00
08 May	400.33	506.67	29 Jun	279.50	295.00
09 May	265.50	291.00	30 Jun	288.00	310.00
10 May	240.00	276.50	09 Jul	291.00	305.00

**Table 10:** The breakpoints used to define sub-indices (standard index for noise measurements).

PSI values	Index category	Equivalent noise levels (dBA)	Noise description
<100	Very good quality	20	Very quiet
100	Good quality	50	Quiet
200	Satisfactory	70	Noisy
300	Unhealthy	90	Very noisy
400	Hazardous	100	Unbearable
500	Seriously Hazardous	130	Seriously Hazardous

## Discussion

The results in this study included the equivalent noise levels and the peak noise levels for daytime and nighttime. The findings were also compared with the World Health Organisation standard of 90 dBs. Equation (1) was applied to the results to obtain the PSI Values.

In the current study, noise levels were collected in a commercial area, where, according to the legislation, noise levels should not exceed 60 dB(A) in an indoor setting and 50 dB(A) in an outdoor setting with windows^20^. The overall results indicate that the noise levels at the two ranks are above the commercial noise regulations^18^ as well as the NIOSH standards^5^. The results indicate that, in both ranks, the noise levels range from 70.3 to 110.2 dB(A). Furthermore, the noise levels were higher in the morning (6:00 to 8:30) and afternoon (16:30 and 18:30). This should be expected as these are rush hour periods. Interestingly, in a study conducted by Khan^1^ in India, traffic noise levels ranged from 85 to 110 dB(A), with the highest measurements obtained in the afternoon (13:00 to 15:00) and evening (17:00 to 19:00). Numerous authors found similar results where road traffic noise levels were higher in commercial places^10,27,28^. In particular, these findings were reported in a study conducted in Namibia, another developing country, where noise pollution levels were higher than recommendations by WHO and National Environment Noise Regulations standard^31^.

Moreover, the findings from this study revealed that the noise levels fall within the extremely dangerous noise sensitivity index. High levels of noise are known to cause annoyance. Several studies report that noise annoyance is associated with, mental health^30^, anger, disappointment, dissatisfaction, withdrawal, helplessness, depression, anxiety, distraction, agitation or exhaustion, and sleep disturbance^31^; risk of hypertension, coronary heart disease, psychological stress and annoyance, and sleep disturbances^10,29,32,33^ in the general population. However, the biggest concern associated with long-term exposure to environmental noise pollution is the resultant noise-induced hearing loss, which has implications for the wellbeing of the individuals exposed to excessive noise, their families and the country at large^34^. Furthermore, according to Chadambuka^35^, 80% of people affected by hearing loss reside in low- and middle-income countries. This has implications for developing countries such as South Africa. For this study in particular, the majority of people exposed to excessive noise constitutes 70% of the general population in South Africa^18^, of which 91.4% are in the low-income group^17^. This has implications for the country as South Africa, as a developing country, is already grappling with high levels of unemployment, low levels of education and a quadruple burden of disease^21–23^. South Africa cannot afford to lose the working class to noise-induced hearing loss, due to environmental noise pollution in the form of road traffic noise.

Although the results of the dosimeter measurements indicated that, except on days falling over paydays or weekends, the noise levels fell within the permissible noise levels recommended by NIOSH. It should be noted that the authors purposefully did not compare the measurements with the recommended standard for commercial noise levels (50–60 dB(A)) as arguably, the noise levels would have been higher. This study made use of the Pollution Standard Index (PSI) to further analyse the data with the aim of quantifying the noise exposure levels to the quality of the noise perceived—very good quality to seriously hazardous quality. The results fell below 300 PSI which is considered a healthy or safe zone. This value (300 PSI) corresponds to approximately 70 dB(A), which is considered safe, yet noisy. According to the US Environmental Protection Agency and the World Health Organization, recommended noise levels below 70 dB(A) over a 24-hour period prevent noise-induced hearing loss, however, noise levels around 70 dB(A) may potentially lead to annoyance^36^. Also, the City of Tshwane Noise Management Policy^24^ states that, at 55–60 dB(A), noise creates annoyance; at 60–65 dB(A), annoyance increases considerably; and above 65 dB(A), constrained behaviour patterns, symptomatic of serious damage caused by noise, arise. While noise levels at 70 dB(A) may not cause a hearing loss, however, the adverse effects associated with noise pollution discussed earlier may be more life-threatening than noise-induced hearing loss. Therefore, it is important to raise awareness about the adverse effects of excessive exposure to noise pollution and potential hearing loss. Raising awareness is in line with the WHO regulations as environmental noise is currently deemed a public health issue globally, but more particularly in developing countries where a study conducted by Onjefu^37^ indicated that traffic noise is the major source of noise pollution. In developing countries, researchers have lamented the fact that noise is not properly recognised^38^ nor are policies on noise regulations strictly enforced in places deemed noisy or surpassing permissible noise levels. This study contributes to the evidence needed to effect these regulations in order to promote prevention and early identification of the auditory and non-auditory effects of excessive noise exposure.

## Conclusion

This study investigated noise levels at two taxi ranks with the aim of quantifying the level of noise and its impact. This was done as the first step in raising awareness about noise pollution and its effects on the health and quality of life in people exposed to excessive noise. Road traffic noise is prevalent in developing countries and, due to urbanisation, commercialisation and mobility, noise pollution is arguably here to stay and cannot be eliminated. As such, raising awareness is the most effective strategy in minimising the short-term as well as the long-term effects of noise pollution on the general population. Raising awareness can be achieved in two ways: (a) having awareness campaigns on noise pollution and its effects; and (b) for the government to implement and enforce regulations and policies on noise pollution, particularly road traffic noise pollution. The onus is on developing countries to be more active in minimising noise pollution as they are the countries that are mostly affected.

## Merits and limitations of the study

To the knowledge of the authors, this is the first study to present quantifiable data to raise awareness on the dangers of excessive noise exposure in a commercial setting in South Africa. Therefore, this study can be used as a springboard for other studies to be conducted with the aim to raise awareness and enforce the implementation of regulation and monitoring of noise in commercial settings. However, it should be noted that data were collected from two taxi ranks and within a limited time period. Therefore, more studies are needed to quantify the noise levels in various commercial areas in order to raise awareness and enforce noise regulations in these settings. This study also used the PSI, a novel pollution modelling tool, to further quantify noise levels at the selected taxi ranks. The use of the PSI was effective as it highlighted the dangers of noise exposures. Although the PSI has been used in other studies to quantify the effects of pollutants in individuals, its merits are not yet conclusive. Therefore, these results need to be interpreted with caution, however, they do highlight the dangers associated with noise pollution in commercial settings.

## Data Availability

All data generated or analysed during this study are included in this published article.

## Abbreviations

NIOSH: National Institute of Occupational Safety and Health
OSHA: Occupational Safety and Health Association
PSI: Pollution Standard Index
SLM: Sound level meter
TWA: Time weighted average
